# The Chinese version of the Myocardial Infarction Dimensional Assessment Scale (MIDAS): Mokken scaling

**DOI:** 10.1186/1477-7525-10-2

**Published:** 2012-01-05

**Authors:** Roger Watson, Wenru Wang, Chantal F Ski, David R Thompson

**Affiliations:** 1 School of Nursing & Midwifery, University of Sheffield, Sheffield, UK; 2 School of Nursing & Midwifery, University of Western Sydney, Sydney, Australia; 3 Alice Lee Centre for Nursing Studies, National University of Singapore, Singapore; 4 Cardiovascular Research Centre, Australian Catholic University, Melbourne, Australia

**Keywords:** cardiac rehabilitation, quality of life, psychometrics, Mokken scaling, Myocardial Infarction Dimensional Assessment Scale

## Abstract

**Background:**

Hierarchical scales are very useful in clinical practice due to their ability to discriminate precisely between individuals, and the original English version of the Myocardial Infarction Dimensional Assessment Scale has been shown to contain a hierarchy of items. The purpose of this study was to analyse a Mandarin Chinese translation of the Myocardial Infarction Dimensional Assessment Scale for a hierarchy of items according to the criteria of Mokken scaling. Data from 180 Chinese participants who completed the Chinese translation of the Myocardial Infarction Dimensional Assessment Scale were analysed using the Mokken Scaling Procedure and the 'R' statistical programme using the diagnostics available in these programmes. Correlation between Mandarin Chinese items and a Chinese translation of the Short Form (36) Health Survey was also analysed.

**Findings:**

Fifteen items from the Mandarin Chinese Myocardial Infarction Dimensional Assessment Scale were retained in a strong and reliable Mokken scale; invariant item ordering was not evident and the Mokken scaled items of the Chinese Myocardial Infarction Dimensional Assessment Scale correlated with the Short Form (36) Health Survey.

**Conclusions:**

Items from the Mandarin Chinese Myocardial Infarction Dimensional Assessment Scale form a Mokken scale and this offers further insight into how the items of the Myocardial Infarction Dimensional Assessment Scale relate to the measurement of health-related quality of life people with a myocardial infarction.

## Introduction

The Myocardial Infarction Dimensional Assessment Scale (MIDAS) is a patient generated disease-specific health status measure for individuals with myocardial infarction. The MIDAS was developed in response to generic instruments failing to measure aspects of myocardial infarction (MI) specific to this patient group e.g. confidence and lifestyle changes [1]. As such, the MIDAS is clinically more sensitive in detecting change following clinical intervention. The questionnaire is also short and simple in format such that it is applicable in a wide range of healthcare settings.

The MIDAS comprises 35 items covering: physical activity, insecurity, emotional reaction, dependency, diet, concerns over medication and side-effects. The MIDAS has good validity and reliability, with Cronbach's α coefficient ranging from 0.74 - 0.95 for the seven domains [1]. The MIDAS has since been translated and validated into Mandarin Chinese (Ch-MIDAS) [2].

Recently, the MIDAS was analysed using Mokken scaling, demonstrating that there was a unidimensional hierarchy within the items [3]. The aim of this study was threefold: 1) to analyse the Ch-MIDAS using Mokken scaling to investigate if there was a unidimensional hierarchical scale in data gathered in China, 2) to compare this with previous Mokken scaling analysis of the MIDAS and 3) to investigate the concurrent validity of the Ch-MIDAS [2] against a Chinese Mandarin translation and validated version of the SF-36 [4]. An explanation of Mokken scaling is provided in the Methods section.

## Methods

This was a secondary analysis of previously collected data [2]: Chinese MI patients (n = 180) completed the Ch-MIDAS which has good validity, reliability and cultural relevance, with Cronbach's α coefficient ranging from 0.74 - 0.94 for the seven domains [2]. The demographics were: males (n = 140); females (n = 40); and mean age 60.6 years (range 36-82, SD = 11.1). The Ch-MIDAS is a 35-item scale with a five option Likert-type response format from 'Never' - 'Always'. Data were entered into the Statistical Package for the Social Sciences (SPSS) version 16.0 and imported into the Mokken Scaling analysis for Polytomous items (MSP) software [5]; a computer programme that searches polytomous data for hierarchical scales using a range of diagnostic criteria. Data were also imported into the 'R' programme version 2.11.1 and, using the Mokken scaling analysis procedure in 'R', and analysed for invariant item ordering (IIO), to be considered below. The SPSS was used to analyse Spearman's rho between the Ch-MIDAS data and the Chinese Mandarin version of the SF-36. Ethical permission was obtained from the universities in Hong Kong and Xi'an where the study was conducted. Written informed consent was obtained from all participants of this study.

### Mokken scaling

Mokken scaling is a method related to item response theory (IRT). Recent publications [6-9] have explained the nature of Mokken scales in non-technical language and also applications of Mokken scaling to HRQoL, activities of daily living and psychological interventions. Mokken scaling is a non-parametric form of IRT. Fundamentally, Mokken scaling seeks unidemensional sets of items from large multivariate datasets. The unique aspect of Mokken scaling is its ability to establish hierarchies of items [10]. Hierarchies of items are useful in psychometrics as they offer additional insight into and utility of scales established using this method. In a Mokken scale, respondents are more likely to endorse low risk items before they endorse high risk items. Thus, the score on a Mokken scale, for example psychological distress, is related to the severity of the psychological distress and, as the score is related to the hierarchy of items, it indicates more precisely the level of the underlying latent trait and, in this case, the level of risk for psychological distress as demonstrated for the General Health Questionnaire [7].

Mokken scaling was used in the present study as opposed to other forms of IRT, for example Rasch modelling, for its less restrictive properties which make it more suitable for comparing different databases [11]. The diagnostics required to evaluate Mokken scaling analyses include H which is used to select items from larger items sets into undimensional item clusters; H > 0.3 indicates weak scale with H > 0.4 indicating a medium scale and H > 0.5 a strong scale with higher values of H indicating greater accuracy in person ordering. The reliability of Mokken scales is estimated using Rho which is a test-retest reliability coefficient with Rho > 0.7 considered to indicate a reliable scale [5]. A Bonferroni method is used to control for Type 1 error rate in testing whether H is positive during the item selection procedure. Monotone homogeneity - an increase in the score on an item as the latent trait increases - can also be estimated using the 'Crit' value - a value generated by the MSP based on a range of criteria [5] - which estimates violations of monotone homogeneity. The MSP was run using the default settings of H > 0.3 and p < 0.05.

The ability to test polytomous scales for IIO has only recently been possible with the advent of the 'R' programme [12]. IIO is considered to be a crucial property of hierarchical scales [13] and, as demonstrated in some recent correspondence, is often misunderstood [14,15]. IIO means, in a scale, 'that the items have the same order with respect to difficulty or attractiveness for all respondents' [[16] p. 578] and the concept has been fully described in a recent paper [17] where the authors also explain how non-intersection of item step response functions (the model of double montonicity) does not necessarily mean that item response functions will not intersect and that testing for IIO using recently developed methods is essential. For the purposes of comparison with previously published work, the items included in a Mokken scale of previous MIDAS data (n = 668) and Ch-MIDAS data from the present study were tested for IIO. The Mokken scaling analysis in R investigates whether item response functions intersect and Htrans (denoted H^T^) is used to investigate the accuracy of the ordering of the resulting item set; values > 0.3 are considered acceptable.

## Results

Table 1 shows the items remaining in the Mokken scale ordered in terms of difficulty (mean score). Fifteen Ch-MIDAS items were retained in a Mokken scale - eight of these in common with those obtained in a previous paper [3] - and a strong, statistically significant and reliable ordering of respondents was obtained (H = 0.52; Rho = 0.94). Items not included in Table 1 showed H values < 0.3. The ordering of items from the most readily to the least readily endorsed runs from 'Felt slowed down' through symptoms of angina to feelings of insecurity and worrying about dying. The scale obtained did not show IIO (H^T ^= 0.26). However, previously published data [3] were run for comparison and these data showed IIO (H^T ^= 0.35). Pearson's correlation between the Ch-MIDAS scores on the Mokken scaled items and the Mandarin Chinese SF-36 was -0.83 (p < 0.001); Pearson's correlation between the total of the original 35 item total for the Ch-MIDAS and the Mandarin Chinese SF-36 was -0.80 (p < 0.001).

**Table 1:** Mokken scaling of Chinese Mandarin version of the MIDAS (n = 180)

Item	Mean	H	Label
20	1.63	0.39	Felt anxious about dying?
23*	1.71	0.44	Felt down or depressed?
25	1.76	0.47	Felt stressed?
18*	1.81	0.46	Felt insecure?
17*	1.88	0.47	Felt vulnerable?
6	2.23	0.52	Been breathless?
7	2.25	0.57	Had chest pain or tightness when undertaking physical activity?
8	2.26	0.56	Felt frustrated at your limitations?
2	2.34	0.48	Had angina symptoms?
11*	2.36	0.53	Felt you cannot perform your domestic duties?
1	2.47	0.57	Thought twice before you undertook physical activity?
9*	2.49	0.54	Needed to rest more?
5*	2.62	0.63	Had no energy?
3*	2.72	0.55	Had angina that affected your life?
4*	2.78	0.60	Felt slowed down?

H = 0.52; Rho = 0.94; p = 0.00012; H^T ^= 0.26; Mean = 33.3 (SD = 12.53); Skewness = 0.21; Kurtosis = -0.95; * = items retained in [3]

## Discussion

Compared with a previous Mokken scaling analysis of the original English version of the MIDAS, there were similarities and differences with the current analysis of the Chinese Mandarin version. Approximately the same number of items was retained in the Mokken scale: 15 in the present analysis as opposed to 14 in the previous analysis and eight of these items were common to both scales. There was a general similarity between the scales in that neither analysis retained items related to medication or diet. There was also a general similarity in the ordering of items with those related to physical aspects (i.e. having 'no energy') being more readily endorsed than items related to psychosocial aspects (e.g. feeling 'insecure'). Moreover, both scales are anchored at the more highly endorsed end for the item 'Felt slowed down'. The items retained in the scale are not the same in the English sample and the Chinese but there are items in common and these could be used to compare the two populations.

The item 'Had angina that affected your life' is more readily endorsed in the Chinese sample and feeling 'down or depressed' much less readily endorsed. This suggests that the Chinese participants prioritise quality of life issues related to their MI differently from the original UK participants. The Mokken scale obtained from UK participants showed IIO. The fact that this was not apparent in the present analysis, suggests that the ordering of Chinese participants' responses to items in the Ch-MIDAS depended on the extent to which their HRQoL was affected by having had a MI. In other words, the ordering of items was not the same at all levels of the latent trait. This difference may be due to the relatively small size of the Chinese sample, however, non-parametric methods such as Mokken scaling can be used with relatively small samples [18]. Lack of IIO does not obviate use of the Ch-MIDAS to order individuals on the basis of the total scale score. Nevertheless, removal of items from any scale, for example in the present analysis on the basis of IIO, can alter the construct validity of the scale composed of the remaining items and full psychometric testing of any instrument newly derived by these methods is necessary.

The Mokken scale obtained from the Ch-MIDAS correlated highly with the Chinese Mandarin translation of the SF-36 indicating that it has concurrent validity when tested against this particular measure of HRQoL. However, further testing against other scales is required to establish construct validity. Hierarchical measurement of latent traits, such as HRQoL, is more useful than simply summing item scores and using the total score alone. With such ordered scales, especially disease specific scales, the deleterious effect of the disease process can be related to specific aspects of HRQoL. For example, someone scoring quite low on the Ch-MIDAS or the MIDAS (e.g. including only having felt slowed down) will have some physical limitations to their HRQoL but someone scoring high and including items related to vulnerability and depression will be more severely affected by their MI. Interventions for people with MI could be tailored on this basis with people scoring low requiring only information about re-mobilisation - with a view to preventing more severe effects on HRQoL. On the other hand, someone scoring high will also require information about re-mobilisation but may also require psychological interventions such as counselling or cognitive behavioural therapy.

## Conclusions

The Ch-MIDAS contains items that show cumulative, hierarchical properties. The scale is broadly similar to that obtained from a UK sample but with some important differences. The present study used data not obtained specifically for the purposes of the present analysis, therefore the study would merit replication with a large sample size.

## List of abbreviations

Ch-MIDAS: Mandarin Chinese translation of the Myocardial Infarction Dimensional: Assessment Scale; H: Loevinger's coefficient; HRQoL: Health related quality of life; IIO: Invariant item ordering; IRT: Item response theory; MIDAS: Myocardial Infarction Dimensional Assessment Scale; MSP: Mokken Scaling analysis for Polytomous items; SF-36: Short Form (36) Health Survey; SPSS: Statistical Package for the Social Sciences.

## Competing interests

The authors declare that they have no competing interests

## Authors' contributions

RW conducted the Mokken scaling analysis and drafted the manuscript. WW participated in the design of the study and collected the data. CS participated in the design of the study and helped to draft the manuscript. DT conceived the study and participated in the design and coordination and helped to draft the manuscript. All authors read and approved the final manuscript.
